# Early-life gut dysbiosis linked to juvenile mortality in ostriches

**DOI:** 10.1186/s40168-020-00925-7

**Published:** 2020-10-12

**Authors:** Elin Videvall, Se Jin Song, Hanna M. Bensch, Maria Strandh, Anel Engelbrecht, Naomi Serfontein, Olof Hellgren, Adriaan Olivier, Schalk Cloete, Rob Knight, Charlie K. Cornwallis

**Affiliations:** 1 Department of Biology, Lund University, Lund, Sweden; 2 Center for Conservation Genomics, Smithsonian Conservation Biology Institute, Washington, DC USA; 3 Department of Pediatrics, University of California San Diego, La Jolla, CA USA; 4 Center for Microbiome Innovation, University of California San Diego, La Jolla, CA USA; 5 Western Cape Department of Agriculture, Directorate Animal Sciences, Elsenburg, South Africa; 6 Western Cape Agricultural Research Trust, Elsenburg, South Africa; 7 South African Ostrich Business Chamber, Oudtshoorn, South Africa; 8 Department of Animal Sciences, Stellenbosch University, Matieland, South Africa; 9 Department of Computer Science & Engineering, University of California San Diego, La Jolla, CA USA; 10 Department of Bioengineering, University of California San Diego, La Jolla, CA USA

**Keywords:** Dysbacteriosis, Gut microbiota, Microbial diversity, Inflammation, Gastrointestinal tract, Disease

## Abstract

**Background:**

Imbalances in the gut microbial community (dysbiosis) of vertebrates have been associated with several gastrointestinal and autoimmune diseases. However, it is unclear which taxa are associated with gut dysbiosis, and if particular gut regions or specific time periods during ontogeny are more susceptible. We also know very little of this process in non-model organisms, despite an increasing realization of the general importance of gut microbiota for health.

**Methods:**

Here, we examine the changes that occur in the microbiome during dysbiosis in different parts of the gastrointestinal tract in a long-lived bird with high juvenile mortality, the ostrich (*Struthio camelus*). We evaluated the 16S rRNA gene composition of the ileum, cecum, and colon of 68 individuals that died of suspected enterocolitis during the first 3 months of life (diseased individuals), and of 50 healthy individuals that were euthanized as age-matched controls. We combined these data with longitudinal environmental and fecal sampling to identify potential sources of pathogenic bacteria and to unravel at which stage of development dysbiosis-associated bacteria emerge.

**Results:**

Diseased individuals had drastically lower microbial alpha diversity and differed substantially in their microbial beta diversity from control individuals in all three regions of the gastrointestinal tract. The clear relationship between low diversity and disease was consistent across all ages in the ileum, but decreased with age in the cecum and colon. Several taxa were associated with mortality (*Enterobacteriaceae*, *Peptostreptococcaceae*, *Porphyromonadaceae*, *Clostridium*), while others were associated with health (*Lachnospiraceae*, *Ruminococcaceae*, *Erysipelotrichaceae*, *Turicibacter*, *Roseburia*). Environmental samples showed no evidence of dysbiosis-associated bacteria being present in either the food, water, or soil substrate. Instead, the repeated fecal sampling showed that pathobionts were already present shortly after hatching and proliferated in individuals with low microbial diversity, resulting in high mortality several weeks later.

**Conclusions:**

Identifying the origins of pathobionts in neonates and the factors that subsequently influence the establishment of diverse gut microbiota may be key to understanding dysbiosis and host development.

Video Abstract

## Introduction

The composition of the microbial community in the gastrointestinal tract of animals (“the gut microbiome”) is extremely important for host fitness and health [1]. Imbalances in the gut microbiome, commonly referred to as gut dysbiosis, have been widely associated with a variety of gastrointestinal and autoimmune diseases such as type 1 diabetes, Crohn’s disease, inflammatory bowel disease, ulcerative colitis, and multiple sclerosis [2–6]. Dysbiosis is typically characterized by loss of beneficial microorganisms, proliferation of pathobionts (opportunistic microorganisms), and a reduction in overall microbial diversity [7, 8]. Transplants of gut microbiota from mice with gastrointestinal disease have been shown to result in similar disease symptoms in recipients, suggesting a strong causal effect of gut dysbiosis on host health [9, 10]. Inflammation of the gastrointestinal tract is often associated with gut dysbiosis, which in turn alters the intestinal mucus layer and epithelial permeability resulting in increased susceptibility to infection, sepsis, and organ failure [11–13].

When and where imbalances in gut microbiota originate is unclear. The diversity and composition of microbes differ markedly across the length of the gastrointestinal tract [14, 15], and it is possible that certain gut regions may act as sources of pathobionts, radiating out to disrupt other parts of the gut. For example, some areas might be more susceptible to pathogenic overgrowth due to low microbial diversity and reduced resilience [16]. Alternatively, dysbiosis may occur throughout the gastrointestinal tract or develop from diverse communities that harbor more pathobionts. Pin-pointing when groups of bacteria start to proliferate in different regions of the gut has been difficult because most studies have used cross-sectional sampling (one sample per individual). As a result, it remains unclear whether bacteria associated with dysbiosis are always present in low abundance, or whether dysbiosis is linked with a sudden influx of foreign microbes from an external source.

An additional problem has been to establish whether certain groups of bacteria are consistently involved in dysbiosis across diverse host species. The vast majority of microbiome studies, and specifically those on dysbiosis, have focused on humans and laboratory mice [7]. This research has shown that certain bacterial taxa seem to be routinely associated with dysbiosis across species and individuals. For example, in inflammatory bowel disease, one of the most common indicators of dysbiosis is elevated levels of *Enterobacteriaceae* (*Gammaproteobacteria*) [10, 17, 18], and a reduction of *Ruminococcaceae* and *Lachnospiraceae* (*Clostridia*) [6, 19]. Whether these patterns extend across more distantly related species and outside laboratory settings is unclear, especially for non-mammalian organisms.

In this study, we examined a novel vertebrate host system, the ostrich (*Struthio camelus*), to understand patterns of gut dysbiosis and its role in the widespread mortality that occurs in captive populations. For example, commercially farmed ostriches suffer from exceptionally high and variable mortality rates during their first 3 months of life [20, 21]. While the causes of mortality are mostly unknown, several candidate pathogens associated with enterocolitis have been reported, for example *Escherichia coli*, *Campylobacter jejuni*, *Pseudomonas aeruginosa*, *Salmonella* spp., *Klebsiella* spp., and multiple *Clostridium* spp. [22–26]. However, whether variation in mortality is due to infection of specific pathogens or the result of microbiome dysbiosis has not yet been established. The studies investigating causes of mortality in ostrich chicks have so far used bacterial culture or species-specific DNA primers [22–26]. These methods can be useful to detect the presence of targeted microorganisms, but searching for a particular culprit may yield ambiguous answers if pathobionts exist in the normal gut microbiota of the host and only exhibit pathogenic tendencies when the community is disturbed [27]. In addition to a high mortality rate, ostriches exhibit large variation in microbial composition between individuals and across gut regions [28]. Because these animals have only been reared in captivity for a very short time relative to other farmed animals (< 120 years) [29], they exhibit several of the advantages of a wild study system (high genetic variation, non-domesticated social groups) while still allowing for controlled conditions and ease of sampling.

Ostrich chicks (*n* = 234) were hatched and raised in four groups under standardized conditions and studied for 12 weeks to investigate gut dysbiosis and mortality patterns. We evaluated the gut microbiota of 68 individuals that died from suspected enterocolitis within 3 months after hatching (referred to as “diseased”) and compared it to 50 individuals that were euthanized as age-matched healthy controls (referred to as “controls”). Age-matched controls were crucial for establishing the characteristics of normal gut microbial communities and how they changed throughout host development. The microbial composition of the ileum, cecum, and colon were characterized to determine the pattern of dysbiosis in different regions of the gastrointestinal tract. Fecal samples collected at 1, 2, 4, and 6 weeks of age from the control and diseased individuals, together with 25 additional individuals that survived the whole period, were analyzed to identify the time point when dysbiosis-related features emerge. Finally, samples from food, water, and soil substrate were examined to evaluate potential sources of dysbiosis-associated bacteria.

## Results and discussion

### Mortality and dysbiosis in different gut regions during ontogeny

**Fig. 1:**
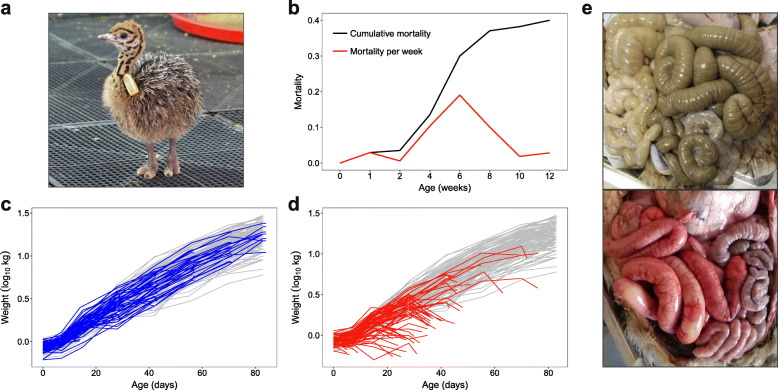
Mortality patterns of ostriches up to 12 weeks of age. **a** One of the ostrich chicks included in the study at 1 week old. **b** The cumulative mortality and mortality rate per week. **c**, **d** Log-transformed weights over time of control individuals that were randomly selected for euthanization at weeks 2, 4, 6, 8, 10, and 12 (blue lines in **c**), and individuals that died of suspected disease (red lines in **d**). Grey lines illustrate weights of all other individuals that survived the whole period. **e** Photographs during dissection illustrating widespread gut inflammation in a diseased individual (bottom) compared to a control individual (top)

Mortality of juvenile ostriches occurred throughout the entire 12-week study period but was highest between 4 and 8 weeks of age, with a peak at 6 weeks (Fig. 1b). Individuals with disease followed the growth curve of all other individuals before rapidly dropping in weight prior to death (Fig. 1c, d). The cause of the weight reduction is unknown, but diseased individuals were observed to stop eating and drinking, and in some cases suffered from diarrhea, so dehydration and wasting are likely explanations. In total, 40% of all chicks died of suspected disease (68/170, excluding 60 controls and 4 injured individuals). Post-mortems of diseased and control individuals revealed that mortality was associated with extensive inflammation of the gastrointestinal tract (Fig. 1e; Figure S1). The gut inflammation scores of diseased individuals (mean ± SD for ileum = 3.1 ± 1.0, cecum = 2.0 ± 1.3, colon = 2.0 ± 1.2) were substantially higher than those of control individuals (ileum = 0.4 ± 1.0, cecum = 0.04 ± 0.29, colon = 0.08 ± 0.45) (Figure S1).

**Fig. 2:**
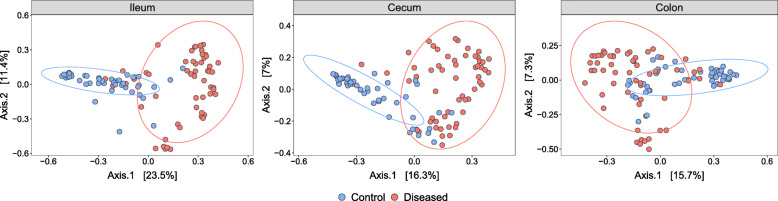
Principal coordinates analysis (PCoA) plots of Bray–Curtis dissimilarities between the microbiomes of control individuals (blue) and diseased individuals (red). Ellipses denote 90% confidence intervals

The structure of the microbiota of diseased and control individuals was extremely different in all three gut regions (Fig. 2, Figure S2, Table 1). Specifically, there were significant differences in the microbial community distances (obtained with both Bray–Curtis (BC) and weighted UniFrac (wUF) measures) between diseased and control individuals, controlling for age, sex, group, and time since death (Table 1). However, Bray–Curtis and weighted UniFrac measures revealed contrasting patterns: Bray–Curtis distances were greatest in the ileum decreasing towards the lower gut (cecum-colon), whereas weighted UniFrac measures were greatest in the colon decreasing towards the ileum (Table 1). Sex, group, and time since death had no significant effects on any of the distance measures of the microbiome in any of the gut regions (Table 1).

**Table 1:** PERMANOVA of microbiome dissimilarities across three gut regions

	Ileum		Cecum		Colon	
	BC	wUF	BC	wUF	BC	wUF
Disease	15.5 ***	8.2 ***	10.8 ***	11.7 ***	7.9 ***	18.2 ***
Age	2.1 *	0.8	5.7 ***	3.0 **	7.1 ***	5.8 ***
Age^2^	1.6 *	0.5	2.8 ***	4.8 ***	3.5 ***	3.1 **
Group	3.1	4.9	2.8	1.9	3.0	2.2
Sex	0.6	0.3	1.1	0.8	1.1	0.8
Time since death	0.8	0.3	0.6	0.5	0.6	0.9

Effect sizes are displayed as *R*^2^ values in percentage with the number of stars indicating level of statistical significance, ***p < 0.001, **p < 0.01, *p < 0.05. *BC* = Bray–Curtis distances, *wUF* = weighted UniFrac distances

Large differences were also found when examining variation in the microbiomes among diseased individuals versus variation among control individuals. The diseased individuals were more similar to each other in the ileal microbiome than the controls were to each other when using Bray–Curtis, but not weighted UniFrac distances (BC Multivariate homogeneity test of group dispersion (betadisper): *F*_1, 99_ = 13.9, *p* = 0.0003. wUF betadisper: *F*_1, 99_ = 0.6, *p* = 0.46) (Figures S3–S4). In contrast, the opposite was true in the cecum and colon (BC cecum betadisper: *F*_1, 105_ = 0.08, *p* = 0.79. BC colon betadisper: *F*_1, 106_ = 1.3, *p* = 0.25. wUF cecum betadisper: *F*_1, 105_ = 11.2, *p* = 0.001. wUF colon betadisper: *F*_1, 106_ = 11.4, *p* = 0.001) (Figure S3). Together, these results show that the bacterial composition of diseased and control individuals differed the most in the ileum, but that the colon contained the most phylogenetically distinct groups.

### Alpha diversity and age-specific dysbiosis in different gut regions

**Fig. 3:**
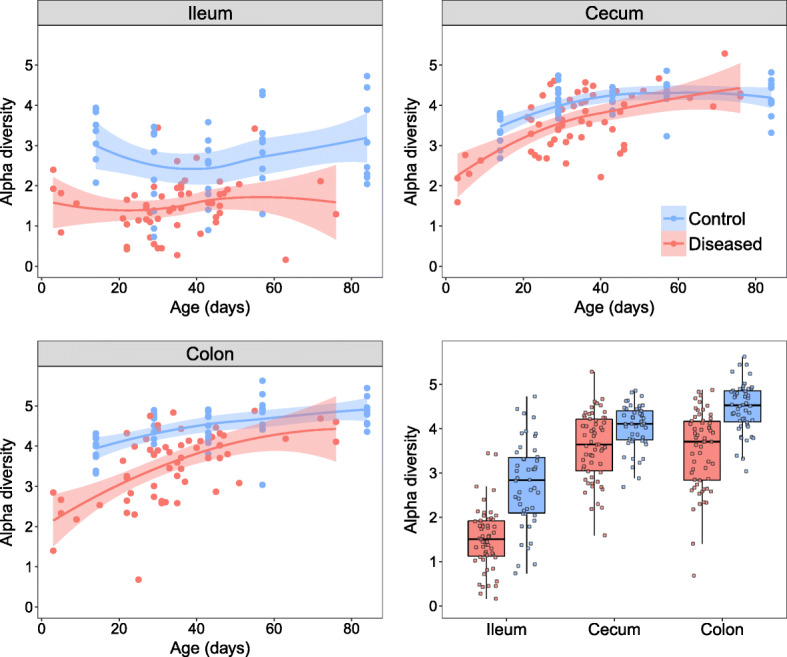
Alpha diversity (Shannon index) during development in the ileum, cecum, and colon. Control individuals are shown in blue and diseased individuals in red. Lines display the fitted local regression smoothing curves and shaded areas the 95% confidence interval. Bottom right panel shows all alpha diversity values together

The microbial alpha diversity of diseased individuals was greatly reduced in all three gut regions in comparison to controls (GLMs disease: ileum *F*_1, 99_ = 56.7, *p* = 2.5e−11; cecum *F*_1, 105_ = 16.1, *p* = 0.0001; colon *F*_1, 106_ = 61.5, *p* = 3.9e−12), controlling for age (Fig. 3). In the ileum, differences persisted across all ages (GLM disease*age: *F*_1, 97_ = 0.0001, *p* = 0.99), and there were little effects of age, even in healthy individuals (GLM age: *F*_1, 98_ = 1.4, *p* = 0.23). In the cecum and colon, diseased individuals had lower alpha diversity than controls at early ages (Table 1; Fig. 3), but these differences diminished with age as diversity generally increased across all individuals (GLM disease*age: cecum *F*_1, 103_ = 10.2, *p* = 0.002; colon *F*_1, 104_ = 9.1, *p* = 0.003). Reductions in alpha diversity associated with disease were therefore evident throughout the gut at early ages, but were restricted to the ileum at older ages (see also [30]).

### Taxa associated with disease in the ileum

**Fig. 4:**
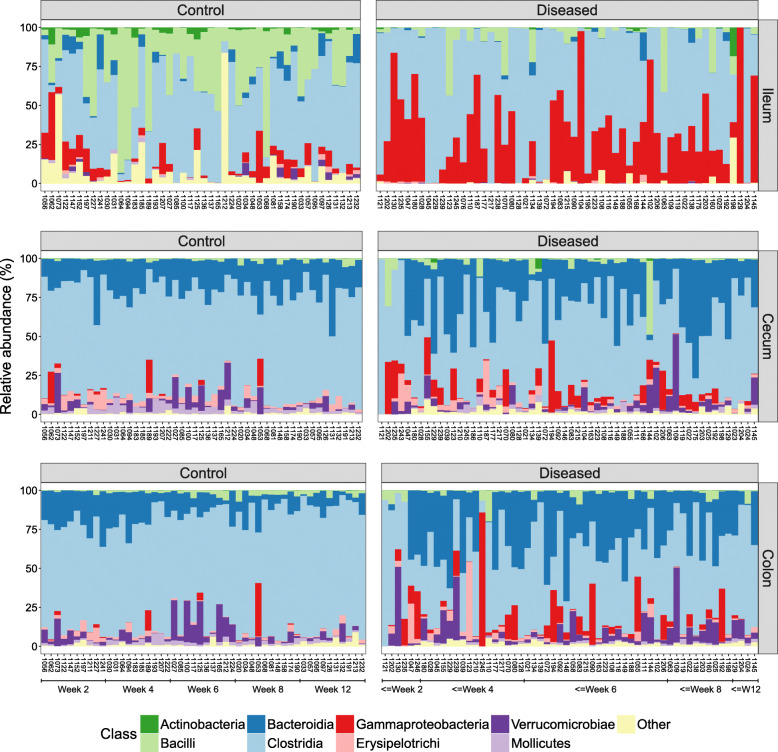
The proportion of bacterial classes per individual and gut region, sorted by age (left bars = youngest, right bars = oldest). Left column = control individuals, right column = diseased individuals. Top row = ileum, middle row = cecum, bottom row = colon

To better understand the microbial dissimilarities between diseased and control individuals, we evaluated the taxonomic composition of all gastrointestinal regions. The ileum showed the most striking evidence of dysbiosis (Fig. 4). Control individuals had a diverse community of different bacterial classes in the ileum, whereas diseased individuals displayed a bloom of *Gammaproteobacteria* and a major reduction in *Bacilli* and other rarer classes. A detailed investigation of the families belonging to *Gammaproteobacteria* showed an almost complete dominance of *Enterobacteriaceae* in the diseased ileum samples, while the control individuals harbored a diverse set of *Gammaproteobacteria* families (Figure S5).

The Gram-negative *Enterobacteriaceae* is a large family that is well-known for encompassing several intestinal pathogens and pathobionts, and is frequently seen in higher abundances in hosts with gut dysbiosis [10, 17, 18]. There were 19 operational taxonomic units (OTUs; sequences with 100% nucleotide identity) associated with *Enterobacteriaceae* in the ileum, and blast searches against the NCBI nucleotide database matched to a wide range of genera, including *Escherichia*, *Klebsiella*, *Shigella*, *Salmonella*, *Yokenella*, *Citrobacter*, *Enterobacter*, *Cronobacter*, *Atlantibacter*, *Pluralibacter*, *Leclercia*, and *Kluyvera*. In previous studies, it has been shown that various members of the *Enterobacteriaceae* family often co-occur and bloom simultaneously during dysbiosis [3, 31], which is consistent with our results.

Another key characteristic of dysbiosis in the ileum was that certain individuals had microbiomes almost entirely comprised of *Clostridia*, a pattern not observed in any control individuals (Figure 4). The families of *Clostridia* showed further striking taxonomic patterns in diseased individuals, including a major increase of *Peptostreptococcaceae* and a marked reduction of *Ruminococcaceae* and other rare families (Figure S5). The *Peptostreptococcaceae* family was represented by six OTUs in our data, and blast searches yielded matches to various species of *Paeniclostridium*, *Paraclostridium*, and *Clostridium*. The most prevalent of these OTUs matched *Paeniclostridium sordellii*, a bacteria known to have virulent strains causing high morbidity and mortality through enteritis and enterotoxaemia in both humans and animals [32, 33].

**Fig. 5:**
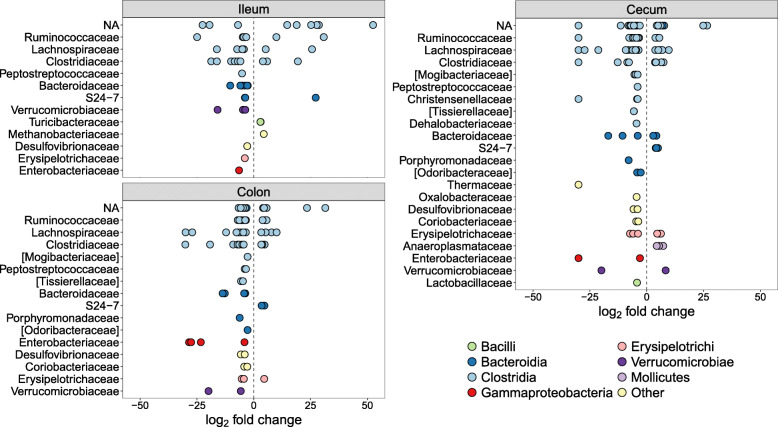
Differentially abundant OTUs (*q* < 0.01) between control and diseased individuals, separate for the three gut regions. *y*-axes show taxonomic families and OTUs have been colored at the class level. Positive log_2_ fold changes indicate higher OTU abundance in the control individuals and negative log_2_ fold changes indicate higher abundance in the diseased individuals. NA = OTUs without family classification

Next, we identified specific OTUs associated with dysbiosis by performing negative binomial Wald tests of bacterial abundances, while controlling for the age of the hosts. Thirty-eight OTUs were significantly overrepresented in the ilea of diseased individuals (Fig. 5), of which most belonged to *Clostridia*, including *Ruminococcaceae*, various *Clostridium* spp., and *Epulopiscium*, but also *Bacteroides*, *Escherichia*, and *Bilophila wadsworthia* (Table S1).

### Taxa associated with disease in the cecum and colon

Examining the relative abundances of bacterial classes in the cecum and colon showed that control individuals were largely similar, exhibiting a relatively stable microbiome composition across hosts and ages. However, there were major disruptions in the microbial composition of both gut regions in diseased individuals (Fig. 4). Similar to the ileum, the *Gammaproteobacteria* were more prevalent in the cecum and colon of diseased individuals, but a reduction in *Clostridia* and an increase in *Bacteroidia* constituted the most prominent differences. Further taxonomic analyses of *Bacteroidia* showed that the family *Porphyromonadaceae* had proliferated in the cecum and colon of diseased individuals (Figure S5). This family encompassed two species in our data, *Parabacteroides distasonis* and *Dysgonomonas* sp., which are commonly found in normal gut microbiota [34]. However, *P. distasonis* has previously been identified as a colitis-promoting species in mice [35] and *Dysgonomonas* members are known to be associated with cachexia and intestinal inflammation [36].

Differential abundance tests identified large similarities in the dysbiosis patterns of the cecum and colon, as 50 out of the 56 (89%) OTUs that were more abundant in the diseased colon samples were also more abundant in the diseased cecal samples (Fig. 5; Tables S2–S3). In addition, 15 out of these OTUs (39%) were also significantly overrepresented in the ileum (Table S1). The most significant OTU in the cecum (*q* = 1.2e−53) and colon (*q* = 2.4e−56) was absent in control individuals but abundant in diseased individuals (Tables S2–S3). This OTU, which was also highly significant in the ileum (*q* = 3.4e−21), had a 100% match against *Clostridium paraputrificum*, a known human pathogen associated with sepsis and necrotizing enterocolitis [37–39]. *C. paraputrificum* has also been experimentally studied in gnotobiotic quails, where it caused lesions and haemorrhages in the gut lining associated with enterocolitis [40].

Besides *C. paraputrificum*, highly significant OTUs that were more abundant in diseased individuals (Tables S2–S3) gave blast matches (99.5–100% identity) to the *Clostridium* species *C. colinum*, *C. cadaveris*, *C. butyricum*, and *C. perfringens*, all of which have previously been linked to acute enterocolitis in both ostriches and other animals [26, 41–44]. Other OTUs that were highly overrepresented in diseased cecal and colon samples belonged to *Enterobacteriaceae*, *Ruminococcaceae*, *Mogibacteriaceae*, *Bacteroides*, *Dorea*, *Sedimentibacter*, *Bilophila wadsworthia*, and *Eggerthella lenta* (Fig. 5; Tables S2–S3). Many of these bacteria constitute part of the normal gut microbiota [45–47] and the majority of all OTUs significantly overrepresented in diseased individuals were also present in some control individuals, albeit at much lower abundances (Tables S2–S3).

### Taxa associated with health in different gut regions

The ileum of diseased individuals showed large reductions in certain bacteria compared to controls (Fig. 4), mainly *Bacilli*, a class in which *Turicibacteraceae* and *Lactobacillaceae* were the most common families. *Turicibacteraceae* included two significant OTUs from *Turicibacter* (Table S1), which showed decreased abundances in diseased ilea. *Turicibacter* has been shown to be highly heritable in humans and mice where it is in direct contact with host cells of the small intestine [48]. This genus has been associated with both health and disease, but is often found to be depleted in animals with diarrhea and enteropathy [49–51].

One of the most striking differences in both the cecum and colon of the diseased individuals was a substantial reduction of the *Bacteroidia* family, *S24-7* (Figure S5). Little is known about *S24-7*, despite it being a prominent component of the normal vertebrate gut microbiota [52]. Nevertheless, studies of mice have reported a potentially beneficial effect of *S24-7*, with abundances often being reduced in diseased hosts [53, 54]. The majority of OTUs with reduced abundances in the colon of diseased individuals were also underrepresented in the cecum (15 out of 19; 79%), indicating large-scale depletion of potentially health-associated bacteria throughout the hindgut. These OTUs belonged to taxa such as *Lachnospiraceae* (e.g., *Coprococcus*, *Blautia*), *Ruminococcaceae* (e.g., *Ruminococcus*), *S24-7*, *Erysipelotrichaceae*, *Clostridium*, *Anaeroplasma*, *Turicibacter*, *Methanobrevibacter*, *Akkermansia muciniphila*, and several unknown *Clostridiales* (Fig. 5; Tables S2–S3).

While 15 OTUs were found to be significantly overrepresented in all three gut regions of diseased individuals, only a single OTU was significantly underrepresented in all gut regions of diseased individuals. This OTU matched the butyrate-producing genus *Roseburia*, which has repeatedly been associated with health. For example, lower abundances of *Roseburia* spp. have been discovered in humans with ulcerative colitis, inflammatory bowel disease, irritable bowel syndrome, obesity, hepatic encephalopathy, and type 2 diabetes [2, 55–57], and in pigs with swine dysentery [58]. These results support the idea that *Roseburia* and many other taxa previously found to be negatively associated with disease, are not only specific to mammalian dysbiosis patterns, but their depletion is a unifying feature of dysbiosis across phylogenetically distant hosts such as humans and ostriches.

### Disruption of the gut microbiota in the weeks preceding death

To establish whether dysbiosis occurs immediately before death or results from imbalances emerging earlier in life, we examined the microbiota of fecal samples that were repeatedly collected prior to death. We found that chick survival up to 4 weeks of age was not related to alpha or phylogenetic diversity of bacteria earlier in life (Table S4). However, the probability of surviving beyond six weeks was predicted by higher alpha diversity at 2 weeks of age (Cox's hazard ratio (HR): 0.57±0.25, *p* < 0.05), but lower alpha diversity at 4 weeks of age (HR: 4.02±0.59, *p* < 0.05), and lower phylogenetic diversity at two and four weeks of age (HR 2 weeks: 1.40±0.15, *p* < 0.05; HR 4 weeks: 1.88±0.24, *p* < 0.01) (Figure S6; Table S4). These results suggest that individuals with low microbial alpha diversity at 2 weeks of age were susceptible to colonization with distinct phylogenetic groups of bacteria, which increased their risk of mortality in the subsequent weeks.

**Fig. 6:**
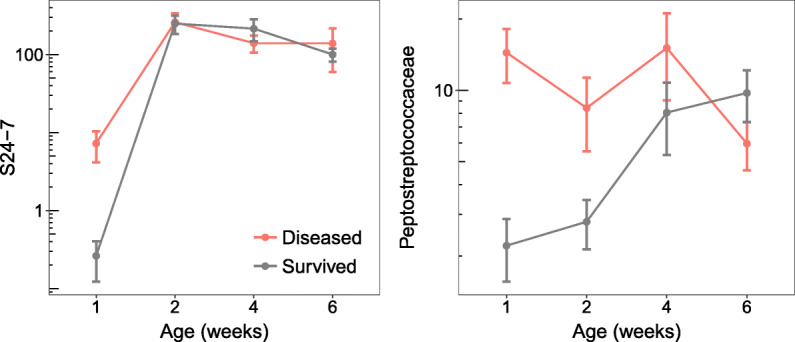
Abundances (normalised and log-transformed) of two bacterial families associated with disease in the weeks preceding death, measured by repeated fecal sampling of individuals. Points and error bars represent means ± SE

Next, we examined if the abundances of bacterial families that differed between diseased and control individuals could predict patterns of future mortality in the weeks leading up to death. There was only weak evidence that having higher abundances of *Lactobacillaceae* at 2 weeks of age and *Turicibacteraceae* at 4 weeks of age had a tendency to positively influence survival (Figure S7; Table S4). The abundances of *Peptostreptococcaceae* and *S24-7* beyond 6 weeks of age were also associated with increased subsequent survival, although not significantly (Table S4). However, there were very strong associations between the abundances of *Peptostreptococcaceae* and *S24-7* during the first week of life and mortality at all subsequent ages, even after controlling for the abundances of these bacterial families at later ages (Peptostreptococcaceae HR range: 1.65±0.13 to 1.73±0.16, all *p* values < 0.001; S24-7 HR range: 1.24±0.11 to 1.60±0.21, all *p* values < 0.05) (Fig. 6; Table S4). This result suggests that the timing of proliferation of certain bacterial groups, such as *Peptostreptococcaceae* and *S24-7*, may be key to host fitness with higher abundances during early ages potentially having detrimental effects even if the same bacterial groups might be beneficial at later ages. It further lends support to the notion that the first couple of days after hatching is a critical period determining whether microbial imbalances ensue, which can lead to increased mortality even months later.

### Environmental sources of gut bacteria

**Fig. 7:**
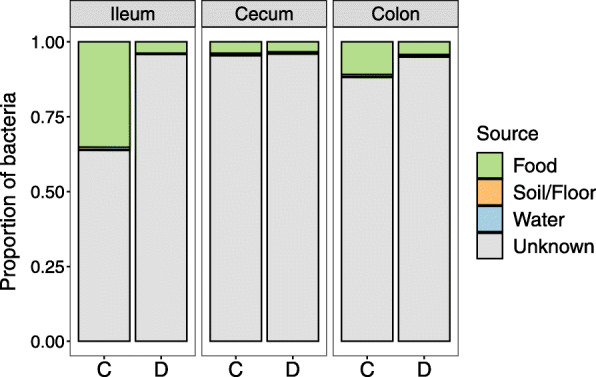
Environmental sources of bacteria present in the different gut sections. C = control individuals and D = diseased individuals

Finally, we evaluated potential environmental sources of the microbes present in the gut of control and diseased individuals. Samples were collected from water, food and soil substrate during the study period and analyzed with SourceTracker [59]. There was essentially no contribution from the water supply (0.1–0.4%) or from the soil (0.2–0.7%) to the gut microbiota of either diseased or control individuals (Fig. 7). Instead, the majority of gut bacteria were from unknown sources (89.9%). Some microbial sequences present in food overlapped with OTUs found in the ileum and colon. However, these were predominantly in control individuals, which may be explained by healthy individuals eating more than sick individuals (Fig. 7). These findings indicate that contaminated food or water were unlikely sources of bacteria associated with mortality.

Our environmental sampling scheme does not exclude the possibility that there are other environmental sources of pathogenic bacteria. For example, several species of wild birds, including cape sparrows, cape weavers, masked weavers, red bishops, and quelea were frequently observed in the chicks’ outdoor enclosures. Sampling water, food, and soil every 2 weeks may also not have been frequent enough to detect potential transient presence of bacteria in the environment or transmission events that may occur sporadically. Nevertheless, our longitudinal fecal microbiome analyses suggest that dysbiosis problems arise early in life from taxa already present in the gut, rather than the sudden acquisition of new taxa. Little is known about the microbiomes of eggs, parents, or the hatching environment for this species, but this is an obvious avenue for future research that may help to identify ways of controlling the prevalence of problematic bacteria during early life. For this study, chicks were reared in isolation from adults because it facilitates management and handling. However, this approach prevents interactions between chicks and parents that may be important for the early establishment of gut microbiota. For instance, coprophagy (feeding on feces) has been shown to be important in the development of microbiota in other animals [60] and ostrich chicks are known to be coprophagic [61]. Providing access to adults (or at least their feces) may allow chicks to seed their microbiome early in life with a balanced and diverse bacterial community, possibly preventing future proliferation of problematic bacteria. This idea, however, remains to be experimentally tested.

## Conclusions

Our study shows that severe disruption of gut bacterial communities is associated with high levels of mortality in developing ostrich chicks. Large-scale shifts in taxon composition, low alpha diversity, and multiple differentially abundant OTUs underlie the dysbiosis pattern seen in diseased individuals. Several taxa associated with disease were disproportionally proliferated in the ileum, cecum, and colon (e.g., *Enterobacteriaceae*, *Peptostreptococcaceae*, *Porphyromonadaceae*, *Clostridium*, *Paeniclostridium*) whereas other taxa were associated with health (e.g., *S24-7*, *Lachnospiraceae* including *Roseburia, Coprococcus* and *Blautia*, *Ruminococcaceae*, *Erysipelotrichaceae*, and *Turicibacter*). Dysbiosis was particularly pronounced in the ileum and in individuals that died at early ages, showing that disruptions to gut microbiota develop in a distinct spatial and temporal manner. The establishment of some of the pathogenic bacteria occurred prior to 1 week of age, which predicted patterns of mortality several weeks later. Yet the rearing environment did not show any evidence of pathogenic sources. A striking feature of the dysbiosis we observed is that many of the implicated harmful and beneficial bacteria have been found to have similar effects in a diverse set of vertebrate hosts, including humans. This pattern suggests that there is a high degree of evolutionary conservatism across some host-microbe associations and that further studies on different vertebrate species may contribute to a general understanding of gut dysbiosis.

## Materials and methods

### Experimental setup

Ostrich eggs were collected over a period of seven days at the Western Cape Department of Agriculture’s ostrich research facility in Oudtshoorn, South Africa and artificially incubated on 19^th^ Aug 2014 to synchronize hatching around 30 September 2014. A total of 234 ostrich chicks hatched and were randomly divided into four groups of approximately 58 chicks each and monitored from day-old until 12 weeks of age. The groups were kept in indoor pens of approximately 4 × 8 m in the same building with access to outdoor enclosures during the day, weather permitting. To reduce potential environmental variation on the development of the gut microbiota, all individuals were reared under standardized conditions with *ad libitum* food and water during daytime. Multiple feeding stations were present in the pens to ensure all chicks could feed freely. The chicks were fed a balanced plant-based pelleted and crumbed diet normally given to ostrich chicks (consisting primarily of corn, soybean, and alfalfa, details in supplementary tables of [30]), and were kept in an area completely separate from adult ostriches. No medicines were given to the chicks during the study period.

### Sample collection

A total of 68 individuals died of suspected enterocolitis during the 12-week period, which we have referred to throughout the text as “diseased.” Many of these chicks exhibited characteristic behavior of sickness shortly before dying (poor appetite, inactivity, listlessness, depressed posture). Additionally, every other week, ten chicks (2–3 individuals from each group) were randomly selected for euthanization and dissection, to act as age-matched controls for the diseased individuals that died. The control individuals were euthanized at 2, 4, 6, 8, 10, and 12 weeks of age by a licensed veterinarian who severed the carotid artery. Four individuals sustained leg or eye injuries and were removed from the study and excluded from all analyses. The contents of the ileum, cecum, and colon of all control and diseased individuals were sampled during dissection and collected in empty 2 ml microtubes (Sarstedt, cat. no. 72.693). To minimize contamination between samples and individuals, lab benches and surfaces were routinely sterilized with 70% ethanol, and dissection equipment was cleaned with hot water, 70% ethanol, and placed in the open flame of a Bunsen burner between each sample collection. During dissections, the time since death (in hours) was recorded (mean time = 6.3 h). When chicks were found dead in the morning, a conservative estimate of time since last checked was given for individuals that were cold (~ 12 h) and 2 h if still warm. Control individuals also varied in time since death because they were euthanized simultaneously and dissected sequentially.

In addition to the intestinal samples, we routinely collected fecal samples from live individuals at 1, 2, 4, 6, 8, 10, and 12 weeks of age. This sampling was conducted on all chicks up to the point of death (diseased and control individuals) and on the chicks that survived the full period (survivors; *n* = 102). Fecal samples were collected in empty 2 ml microtubes 1 day before scheduled euthanizations of control individuals took place. Weight measurements of all individuals were obtained at hatching, during each fecal collection event and immediately prior to dissection. Environmental samples were collected throughout the experiment by wetting sterile cotton swabs in phosphate-buffered saline (PBS) and swabbing food, drinking water, and the soil/floor of the ostrich chicks’ enclosures during each sampling event. All samples were frozen at − 20°C after collection.

During dissections, photographs of the gastrointestinal tract of each individual were taken and later scored for inflammation using a four-point scale: 0 = no visible inflammation, 1 = minor inflammation, 2 = intermediate inflammation, 3 = major inflammation, and 4 = extreme and severe inflammation. The author (E.V.) performing the inflammation assessment was blind to whether individuals had been euthanized or died (control/diseased). Twenty-three measures (7% of 323) were given a score of NA because it was not possible to assess the inflammation (e.g., gut region not properly visible on photograph) (Table S5).

### DNA sequencing

We prepared sample slurries based on the protocol in [62] and extracted DNA using the PowerSoil-htp 96 well soil DNA isolation kit (Mo Bio Laboratories, cat no. 12955-4) as recommended by the Earth Microbiome Project (www.earthmicrobiome.org). Libraries were prepared for amplicon sequencing of the V3 and V4 regions of the 16S rRNA gene using Illumina fusion primers containing the target-specific primers Bakt_341F and Bakt_805R [63] according to the Illumina 16S Metagenomic Sequencing Library Preparation Guide (Part # 15044223 Rev.B). The samples were sequenced as 300 bp paired-end reads over three sequencing runs on an Illumina MiSeq at the DNA Sequencing Facility, Department of Biology, Lund University, Sweden. We sequenced a total of 323 ileum, cecum, and colon samples from all individuals that died (*n* = 68) and euthanized (control) individuals at 2, 4, 6, 8, and 12 weeks of age (*n* = 50 in total; 10 individuals per week, excluding samples taken at 10 weeks of age due to the limited number of deaths of diseased individuals at this time point; Table S5). We also sequenced a total of 378 fecal samples from weeks 1, 2, 4, and 6: 181 from the diseased individuals, 99 from control individuals, and 98 from survivors (Table S6). The sequence data from fecal samples of control individuals and survivors have been used in a previous study, which evaluated the maturation of fecal microbiomes in healthy chicks during the full 3-month period [30]. Finally, we sequenced 24 environmental samples (8 food, 8 water, 8 soil) during weeks 2, 4, 6, and 8, and 4 negative samples (blanks) (Table S5).

### Data processing

Primers were removed from reads using Trimmomatic (v. 0.35) [64] and quality-filtered using the script multiple_split_libraries_fastq.py in QIIME (v. 1.9.1) [65]. Bases with a Phred score < 25 at the 3′ end of reads were removed and samples multiplexed. Forward reads were retained for downstream analyses due to lower base quality in reverse reads. Amplicon sequence variants (ASVs) were clustered in Deblur (v. 1.0.0) [66] and assigned using the RDP classifier (v. 2.2) [67]. ASVs are referred to as operational taxonomic units (OTUs) in this study to aid consistency with previous ecological and evolutionary research. In Deblur, the minimum reads option was set to 0 to disable automatic filtering and all sequences were trimmed to 220 bp. We used the OTU table produced after both positive and negative filtering, which removes reads containing PhiX or adapter sequences, and only retains 16S sequences. PCR-originating chimeras are filtered inside Deblur by default [66]. We removed all OTUs that were either classified as mitochondria or chloroplasts, present in the negative samples, only appeared in one sample, or with a total sequence count of less than 10. We further filtered out all samples with a total sequence count of less than 500, resulting in 7 ileal and 3 environmental samples being excluded. Average read count per OTU was 1005.9 for the intestinal samples and 944.2 for the fecal samples.

### Data analyses

All statistical analyses were performed in R (v. 3.3.2) [68], and all plots were made using ggplot2 [69]. A phylogenetic tree for the UniFrac and phylogenetic diversity measures was made with FastTree [70]. Bray–Curtis and weighted UniFrac [71] distances between microbiomes were calculated in phyloseq (v. 1.19.1) [72] and examined using a PERMANOVA with the adonis function in vegan (v. 2.4-2) [73]. Age effects on the microbiome were evaluated by fitting a linear term and a quadratic age term with *Z*-transformed values. Beta diversity was tested with a multivariate homogeneity of groups dispersions test using the betadisper function in vegan [73]. We calculated alpha diversity using Shannon’s *H* index and phylogenetic diversity using Faith’s weighted abundance of phylogenetic diversity. Variation in diversity was analyzed using a GLM with a Gaussian error distribution, health status (control versus diseased), age, and their interaction as fixed effects. Separate GLMs were used for each gut region.

To evaluate bacterial abundances, we first modelled counts with a local dispersion model and normalised per sample using the geometric mean, according to DESeq2 [74]. Differential OTU abundances between control and diseased individuals were subsequently tested in DESeq2 with a negative binomial Wald test while controlling for the age of individuals and with the beta prior set to false [74]. Results for the specific comparisons were extracted (e.g., “ileum control” versus “ileum diseased”) and *p* values were corrected with the Benjamini and Hochberg false discovery rate for multiple testing. OTUs were considered significantly differentially abundant if they had an adjusted *p* value (*q* value) < 0.01. Environmental samples were analyzed with SourceTracker [59].

To estimate the ages at which diversity and bacterial taxa predicted survival, we analyzed the fecal samples using Cox Proportional Hazards models in the R package survival (v. 2.44-1.1) [75]. These models examine whether explanatory variables are associated with a greater risk (beta coefficient > 1) or lower risk (beta coefficient < 1) of mortality. Separate models were fitted for each measure of diversity and each bacterial family with their measurements at weeks 1, 2, 4, and 6 fitted as explanatory variables (Table S7). Later ages than week 6 were not included as there was little variation in mortality after this time (Fig. 1). Because individuals that died very early in life had missing data for later time points, it was not possible to include all explanatory variables simultaneously without restricting the data to individuals that survived past week 6. Therefore, measures from each age were sequentially entered into models in a chronological order (e.g., week 1 followed by week 1 and 2). By doing this, we were able to test how microbiome features at week 1 predicted survival past week 1, how microbiome features at week 2 predicted survival past week 2, while controlling for any microbiota differences at week 1, and so forth.

## Supplementary information

**Additional file 1.** Supplementary figures.

**Additional file 2.** Supplementary Table S1. Significant differentially abundant OTUs in the ileum.

**Additional file 3.** Supplementary Table S2. Significant differentially abundant OTUs in the caecum.

**Additional file 4.** Supplementary Table S3. Significant differentially abundant OTUs in the cecum.

**Additional file 5.** Supplementary Table S4. Predicting patterns of mortality from fecal microbiota during preceding ages using Cox proportional hazard models.

**Additional file 6.** Supplementary Table S5. Intestinal and environmental samples metadata.

**Additional file 7.** Supplementary Table S6. Fecal samples metadata.

**Additional file 8.** Supplementary Table S7. Survival analysis.

## Data Availability

Supporting information has been made available online in association with this paper. Sequences have been uploaded to the European Nucleotide Archive at EMBL-EBI under accession numbers: PRJEB28512 (fecal samples) and PRJEB28515 (intestinal and environmental samples).
