# Supplementary material for: Early-life gut dysbiosis linked to juvenile mortality in ostriches
Source: Microbiome. 2020 Oct 12;8:147. doi: 10.1186/s40168-020-00925-7 (PMC7552511; doi:10.1186/s40168-020-00925-7)
Supplement: Supplementary file 2 — Additional file 1. Supplementary figures. [file 40168_2020_925_MOESM1_ESM.pdf]

Supplementary Figures

Early-life gut dysbiosis linked to juvenile mortality in ostriches

Elin Videvall, Se Jin Song, Hanna M. Bensch, Maria Strandh, Anel Engelbrecht, Naomi Serfontein, Olof Hellgren, Adriaan Olivier, Schalk Cloete, Rob Knight, Charlie K. Cornwallis

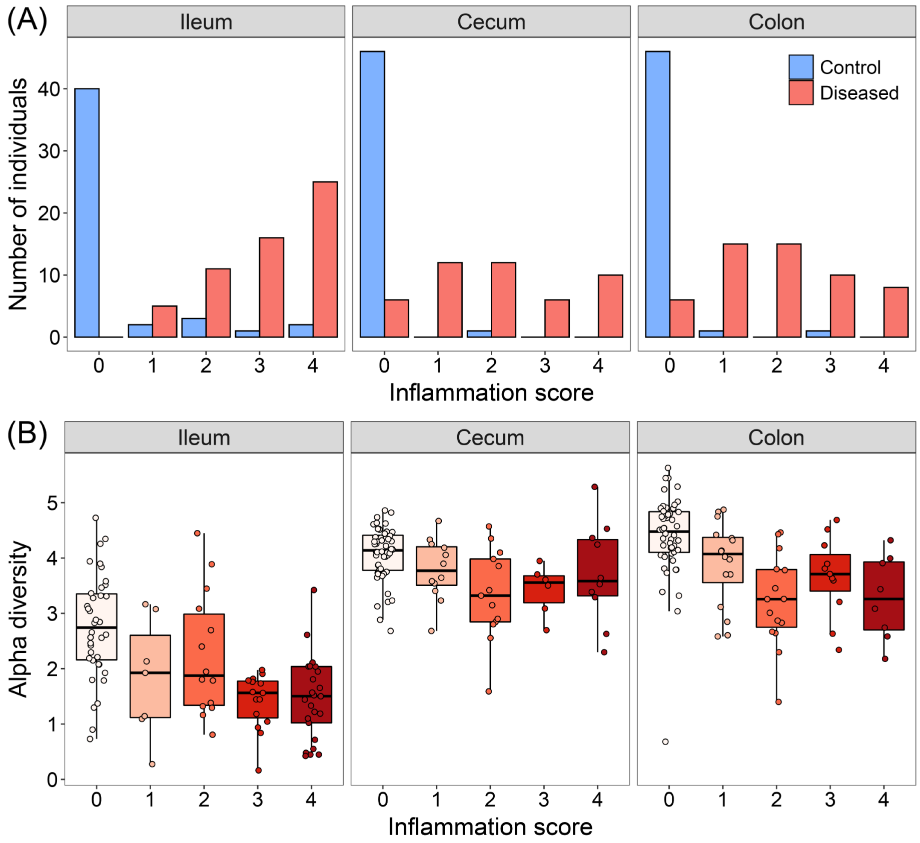

Figure S1. Gut inflammation patterns in ostrich juveniles. (A) Number of control and diseased host individuals and (B) alpha diversity values of diseased individuals, in relation to their respective gut inflammation scores. Inflammation score is based on visible redness of the gut region, where 0 indicates no redness and 4 indicates extensive and major inflammation (see Methods section in the paper).

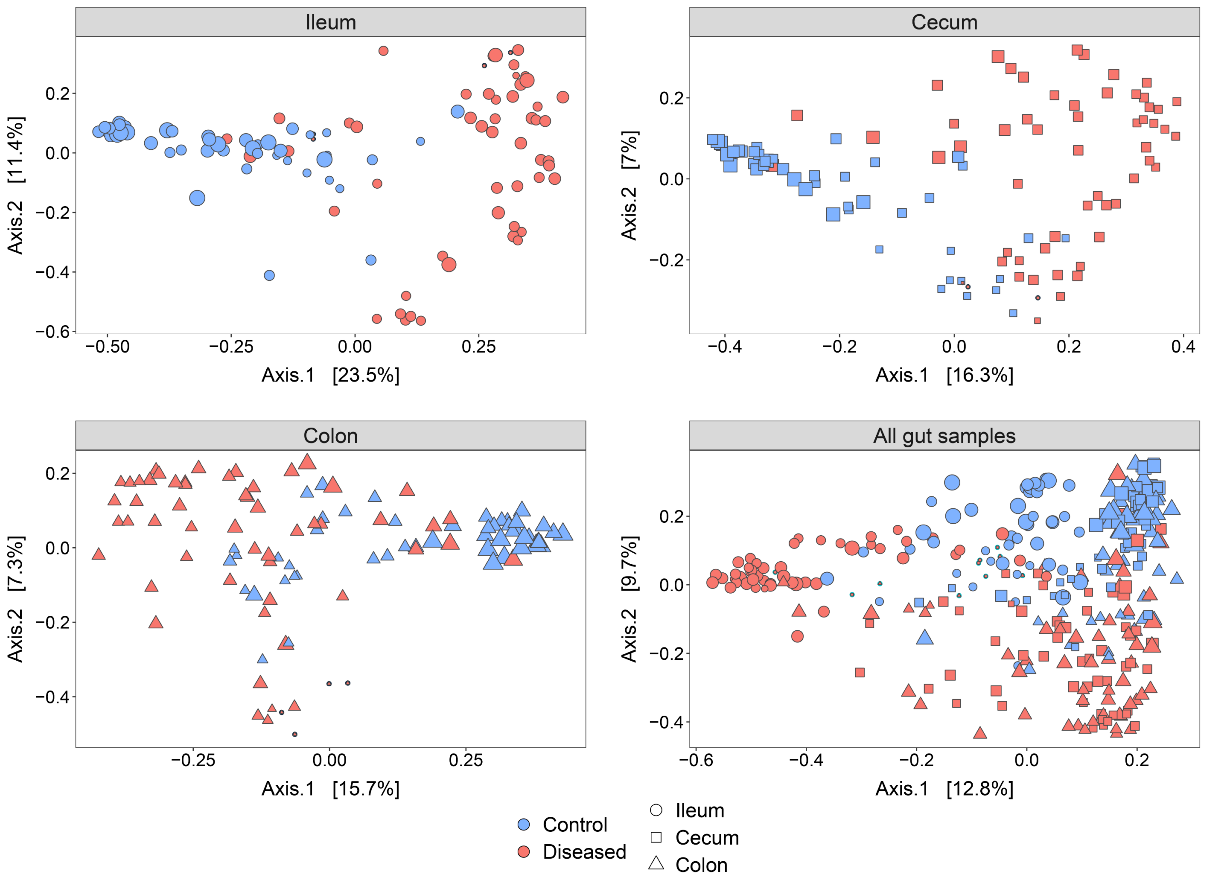

Figure S2. Principal Coordinates Analysis (PCoA) plots of Bray-Curtis dissimilarities displaying the first two dimensions of the microbiota variation between samples. Control individuals are given in blue, diseased individuals in red, and the symbols illustrate gut region. Size of symbols indicates age of individuals, with the smallest size = week 0, and the largest = week 12.

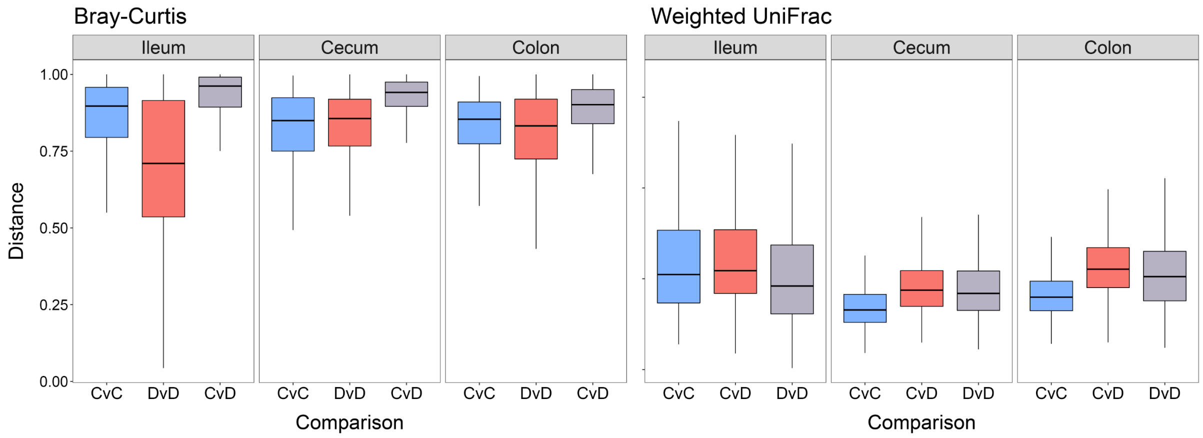

Figure S3. Bray-Curtis and weighted UniFrac distance comparisons between microbiome samples. CvC = control individuals vs. other control individuals, DvD = diseased individuals vs. other diseased individuals, and CvD = control individuals compared to diseased individuals.

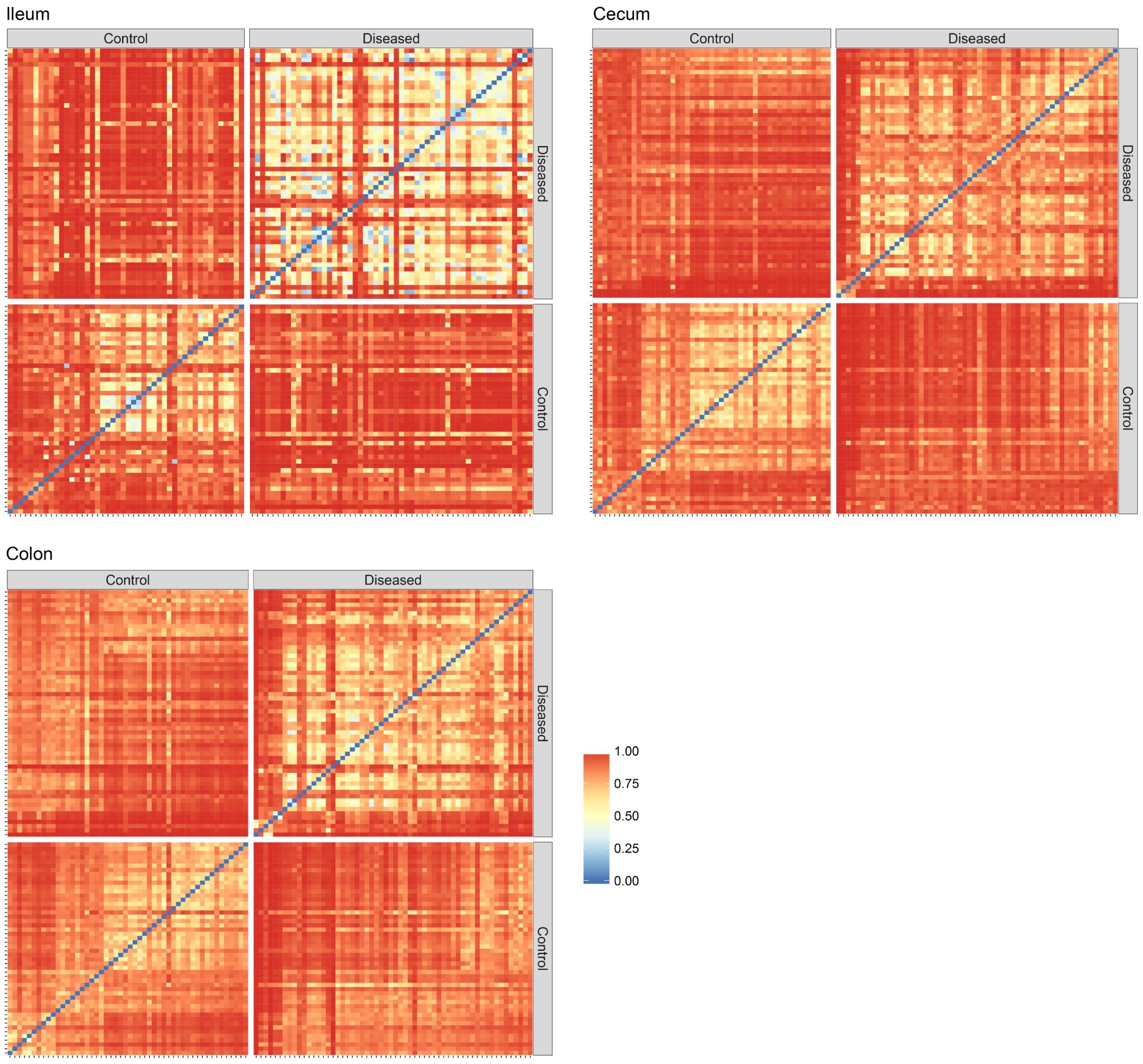

Figure S4. Heatmaps of Bray-Curtis distances between the gut microbiomes of all individuals, separate for the ileum, cecum, and colon samples, sorted by age (youngest to the left). Warmer colors signify greater dissimilarities.

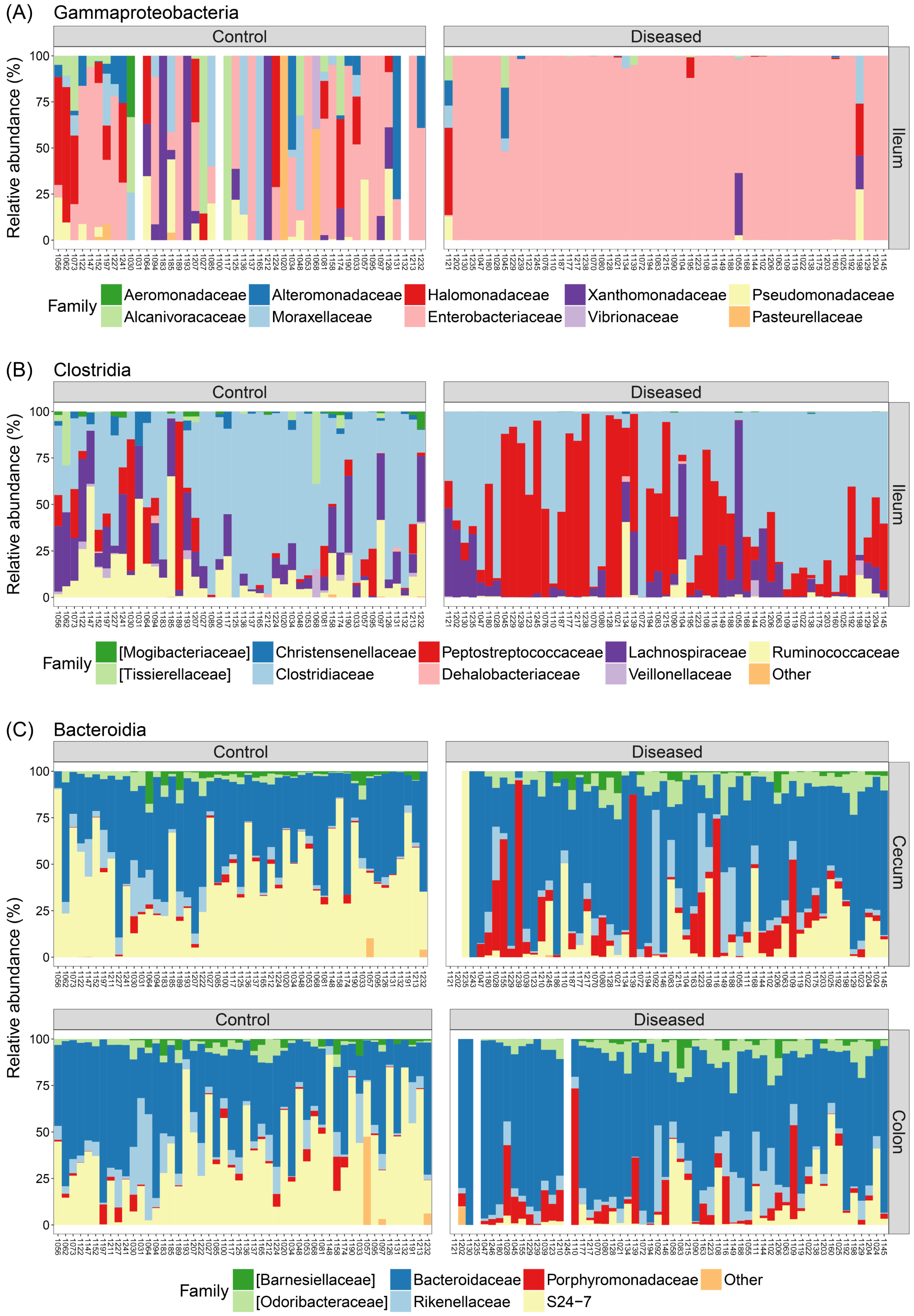

Figure S5. The proportion of three bacterial families in the gut microbiota. (A) *Gammaproteobacteria* in the ileum, (B) *Clostridia* in the ileum, and (C) *Bacteroidia* in the cecum and colon. x-axes show individuals, sorted by age. White bars denote individuals without any members of that particular class.

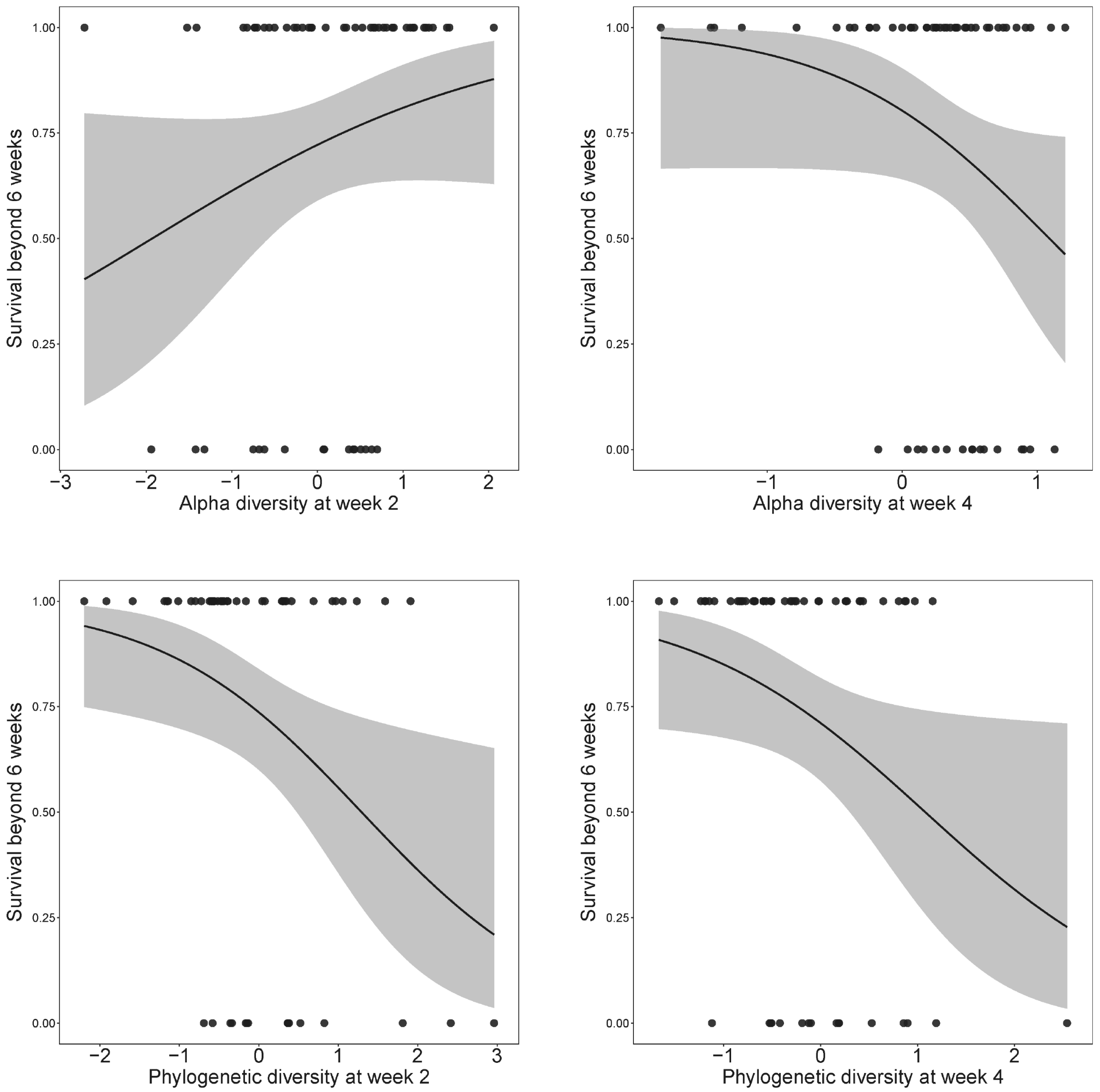

Figure S6. The effect of alpha diversity and phylogenetic diversity on the probability of survival beyond six weeks of age. These plots are based on data from fecal microbiomes continuously sampled during development.

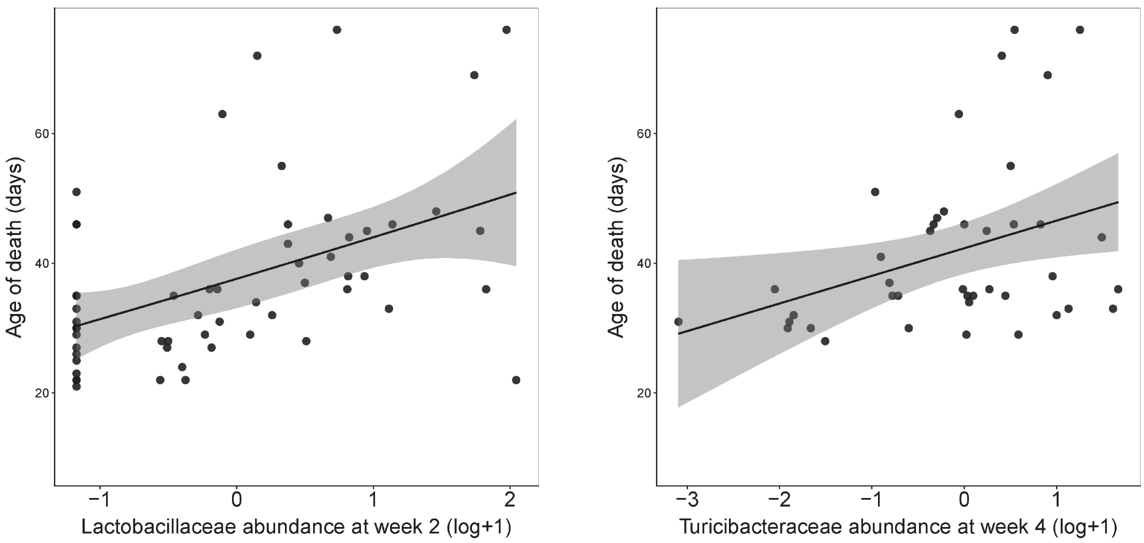

Figure S7. The relationship between normalized abundances of *Lactobacillaceae* and *Turicibacteraceae* in fecal microbiomes at week 2 and 4, respectively, and the age at which chicks died. These bacterial families were found to have different relative abundances between diseased and healthy individuals.
