# Supplementary material for: Early-life gut dysbiosis linked to juvenile mortality in ostriches
Source: Microbiome. 2020 Oct 12;8:147. doi: 10.1186/s40168-020-00925-7 (PMC7552511; doi:10.1186/s40168-020-00925-7)
Supplement: Supplementary file 6 — Additional file 5. Supplementary Table S4. Predicting patterns of mortality from fecal microbiota during preceding ages using Cox proportional hazard models. [file 40168_2020_925_MOESM5_ESM.pdf]

Table S4. Predicting patterns of mortality from fecal microbiota during preceding ages using Cox proportional hazard models. Regression coefficients ± SEs are given and significant predictions are highlighted in bold font (*P < 0.05, **P < 0.01, ***P < 0.001).

**Analysis**	**Age at fecal sampling (weeks)**	**Age at death (>weeks)**			
		**2**	**4**	**6**	**8-12**
Alpha diversity	1	0.81±0.14	0.85±0.13	0.93±0.17	0.91±0.27
	2	-	0.79±0.13	0.91±0.16	**0.57±0.25***
	4	-	-	0.91±0.14	**4.02±0.59***
	6	-	-	-	0.64±0.28
Phylogenetic diversity	1	0.92±0.13	0.94±0.13	0.97±0.16	1.05±0.25
	2	-	1.24±0.13	**1.40±0.15***	**1.88±0.24****
	4	-	-	1.39±0.18	**1.93±0.31***
	6	-	-	-	1.27±0.23
Enterobacteriaceae	1	1.20±0.15	1.20±0.15	1.16±0.19	1.42±0.32
	2	-	1.04±0.13	0.93±0.17	0.60±0.29
	4	-	-	1.30±0.15	1.07±0.25
	6	-	-	-	1.45±0.27
Peptostreptococcaceae	1	**1.72±0.13*****	**1.65±0.13*****	**1.73±0.16*****	**1.72±0.26***
	2	-	1.06±0.13	0.79±0.19	1.03±0.30
	4	-	-	1.13±0.16	0.53±0.36
	6	-	-	-	1.20±0.30
Ruminococcaceae	1	0.78±0.14	0.82±0.15	0.94±0.19	1.00±0.35
	2	-	0.88±0.14	0.94±0.17	1.62±0.36
	4	-	-	1.13±0.16	1.66±0.37
	6	-	-	-	1.50±0.30
Porphyromonadaceae	1	1.08±0.12	1.14±0.13	1.10±0.15	0.97±0.24
	2	-	0.80±0.13	0.83±0.16	0.96±0.25
	4	-	-	1.15±0.17	0.80±0.31
	6	-	-	-	1.41±0.29
Clostridiaceae	1	0.97±0.14	0.96±0.14	0.94±0.17	0.79±0.26
	2	-	0.92±0.14	1.02±0.16	1.03±0.30
	4	-	-	0.93±0.14	1.11±0.26
	6	-	-	-	0.98±0.27
Lactobacillaceae	1	1.14±0.10	1.21±0.11	1.11±0.19	1.20±0.25
	2	-	0.74±0.14	0.88±0.17	1.35±0.27
	4	-	-	0.94±0.16	0.94±0.81
	6	-	-	-	1.03±0.92
Turicibacteraceae	1	0.91±0.14	0.89±0.15	0.93±0.18	1.06±0.29
	2	-	1.11±0.14	1.04±0.17	0.83±0.31
	4	-	-	0.74±0.16	1.19±0.30
	6	-	-	-	0.83±0.25
S24-7	1	1.22±0.11	**1.24±0.11***	**1.35±0.12***	**1.60±0.21***
	2	-	0.97±0.13	0.99±0.15	0.63±0.26
	4	-	-	1.17±0.18	1.29±0.31
	6	-	-	-	0.99±0.27
Lachnospiraceae	1	1.04±0.14	1.06±0.14	1.12±0.17	0.92±0.32
	2	-	1.10±0.15	0.93±0.19	0.62±0.40
	4	-	-	1.00±0.15	1.38±0.24
	6	-	-	-	1.46±0.24
